# CUEING EFFECT ON DUAL TASK COORDINATION AMONG YOUNGER AND OLDER ADULTS

**DOI:** 10.1093/geroni/igac059.789

**Published:** 2022-12-20

**Authors:** Yue Hu, Helene Fung

**Affiliations:** The Chinese University of Hong Kong, Hong Kong, Hong Kong; The Chinese University of Hong Kong, Hong Kong, Hong Kong

## Abstract

Dual task coordination, which refers to the ability to coordinate the cognitive processes involved in performing two tasks with a temporal overlap, is evident in many if not all situations in the daily life of the older adults. The dual-task performance of older adults has been shown to be associated with driving performance, risk of falls, risk of car accidents and the absence of mild cognitive impairment. However, some researchers found that healthy older adults had worse dual-task performance than their younger counterparts, whereas other researchers found no age difference in dual-task performance. To address the mixed findings, the present work examined the effect of cue words on dual task coordination based on the selection, optimization, and compensation model. A total of 65 younger adults (24 females, mean age 21.80±1.99) and 91 older adults (36 females, mean age 65.38±5.61) were recruited via the platform Prolific. All participants judged the orientation of two arrows displayed with a shorter or longer temporal overlap. Before the onset of the first arrow, “difficult”, “easy” or a blank screen occurred on the computer screen. A significant cue effect was detected among younger adults regardless of the cue word. Yet, older adults had better dual-task performance under the condition of “difficult” cue word as compared with the other conditions. The findings highlight the potentially crucial role of higher expected task difficulty in preparing older adults to compensate for their age-related decline in dual-task coordination.

